# Expression of Olfactory Signaling Genes in the Eye

**DOI:** 10.1371/journal.pone.0096435

**Published:** 2014-04-30

**Authors:** Alexey Pronin, Konstantin Levay, Dmitry Velmeshev, Mohammad Faghihi, Valery I. Shestopalov, Vladlen Z. Slepak

**Affiliations:** 1 Department of Molecular and Cellular Pharmacology, University of Miami School of Medicine, Miami, Florida, United States of America; 2 Department of Psychiatry & Behavioral Sciences, University of Miami School of Medicine, Miami, Florida, United States of America; 3 Bascom Palmer Eye Institute, University of Miami School of Medicine, Miami, Florida, United States of America; Cedars-Sinai Medical Center; UCLA School of Medicine, United States of America

## Abstract

**Purpose:**

To advance our understanding how the outer eye interacts with its environment, we asked which cellular receptors are expressed in the cornea, focusing on G protein-coupled receptors.

**Methods:**

Total RNA from the mouse cornea was subjected to next-generation sequencing using the Illumina platform. The data was analyzed with TopHat and CuffLinks software packages. Expression of a representative group of genes detected by RNA-seq was further analyzed by RT-PCR and *in situ* hybridization using RNAscope technology and fluorescent microscopy.

**Results:**

We generated more than 46 million pair-end reads from mouse corneal RNA. Bioinformatics analysis revealed that the mouse corneal transcriptome reconstructed from these reads represents over 10,000 gene transcripts. We identified 194 GPCR transcripts, of which 96 were putative olfactory receptors. RT-PCR analysis confirmed the presence of several olfactory receptors and related genes, including olfactory marker protein and the G protein associated with olfaction, Gαolf. *In situ* hybridization showed that mRNA for olfactory marker protein, Gαolf and possibly some olfactory receptors were found in the corneal epithelial cells. In addition to the corneal epithelium, Gαolf was present in the ganglionic and inner nuclear layers of the retina. One of the olfactory receptors, Olfr558, was present primarily in vessels of the eye co-stained with antibodies against alpha-smooth muscle actin, indicating expression in arterioles.

**Conclusions:**

Several species of mRNA encoding putative olfactory receptors and related genes are expressed in the mouse cornea and other parts of the eye indicating they may play a role in sensing chemicals in the ocular environment.

## Introduction

The ocular surface is continuously exposed to tear components, xenobiotics, microorganisms and their metabolites. Recognition of these environmental factors is essential for protection of the eye from infection and maintaining homeostasis. Unnecessary activation of immune defenses, for example, in response to a harmless commensal bacterium, could trigger inflammation, leading to opacity of the cornea and possible loss of vision.

Currently, it is thought that the tasks of sensing danger signals, discrimination of pathogens from commensals and initiating immune responses are mediated by toll-like receptors (TLRs) that are abundant on the ocular surface [1], [2]. TLRs are an evolutionarily conserved family of 13 proteins that bind to common molecules associated with infection such as bacterial cell wall lipopolysaccharides, certain RNA and DNA and material from damaged host cells, *e.g.* heat shock proteins [3]. Accordingly, TLRs are referred to as pattern recognition receptors.

Unlike TLRs, most G protein-coupled receptors (GPCRs) are highly selective, *e.g.*, a receptor for norepinephrine will not recognize dopamine, which is different only by a single hydroxyl group. GPCRs are encoded by hundreds of distinct genes, many of which are expressed in tissue- and cell-specific manner [4]. These features benefit pharmacologic intervention, and targeting GPCRs in the past decades brought about revolutionary success in many areas, for example, neuropharmacology and treatment of cardiovascular diseases [5], [6]. However, little was done to target corneal GPCRs, mainly due to insufficient information about this class of receptors in the cornea. A few common receptors, e.g., muscarinic [7] and adrenergic [8], [9] were found in the cornea and conjunctiva and shown to play a role in wound repair via regulation of adenylyl cyclase and MAP kinase. Receptors of such lipid mediators as platelet-activating factor and lipoxin A4 exert powerful effects on wound healing, inflammation and other processes on in the cornea [10], [11].

The most numerous class of GPCRs in vertebrates is the family of olfactory receptors (Olfrs). This family is predicted to contain approximately 1,100 functional genes in the mouse [12]. Ligands have been identified for fewer than 100 of these receptors [13], with the majority of genes assigned to the Olfr family continuing to be orphan receptors. Interestingly, it was recently found that some of these genes are expressed outside of the nasal cavity and serve functions different from olfaction - in the heart, kidney, skeletal muscle, prostate and sperm [14]. Along with taste receptors, Olfrs are often referred to as chemosensory receptors, reflecting the idea they may participate in physiological functions other than senses of smell and taste.

Here, we investigated the hypothesis that GPCRs may participate in the chemosensory function of the ocular surface. As the first step, we sequenced a mouse corneal transcriptome and found mRNA encoding many GPCR genes including putative Olfrs. We also found mRNA encoding related proteins such as olfactory G protein Gαolf and olfactory marker protein (OMP). Our subsequent analysis showed expression of several olfaction-associated genes not only in the cornea but in other eye tissues too. These results indicate that olfactory signaling genes may play a role in sensing the environment and maintaining homeostasis of the eye.

## Materials and Methods

### Animals

All procedures with the mice used in this study were performed according to the Guidelines for the Care and Use of Laboratory Animals of the National Institutes of Health and the protocol approved by the University of Miami Committee on Use and Care of Animals (Protocol Number: 14-016). All efforts were made to minimize animal suffering. Prior to isolation of their eyes, C57BL/6 mice 8–10 weeks of age were euthanized by CO_2_ inhalation followed by the cervical dislocation.

### Isolation of Total RNA and Next-generation Sequencing

Corneas were removed from the eyes by excision. Corneal tissue was immediately homogenized in Trizol reagent (Invitrogen) and the total RNA was isolated using Direct-zol RNA Miniprep kit according to the recommended protocol (Zymo Research). To eliminate potential contamination with genomic DNA (gDNA) the samples were subjected to additional treatment with recombinant DNase I (Ambion) for 30 min at 37°C and the total RNA was re-purified over RNeasy mini columns (Qiagen). Quality and the RNA concentration in the samples were evaluated using the RNA chip bioanalyzer (Agilent Technologies). As an additional step to test RNA quality, we performed RT-PCR assays using a panel of primers for several ubiquitously expressed genes including *Gapdh* and G protein subunits (*Gnb1*, *Gnai2*). Samples were depleted of both cytoplasmic and mitochondrial ribosomal RNA using Ribo-Zero-Gold kit (Epicentre). Library preparation for next generation sequencing was performed using Illumina TrueSeq library preparation kit that allows for paired-end directional RNA sequencing. Next-generation sequencing of corneal RNA was performed at the Hussman Institute for Human Genomics core facility using the Illumina HiSeq 2000 platform.

### Analysis of the NGS Results

Raw reads from Illumina HiSeq platform were subjected to quality control and then trimmed of library adapters using a custom Python script. Trimmed reads were then aligned with TopHat version 2.0.9 [15], [16] to the mouse genome mm10. *Ab initio* assembly of aligned reads was performed with CuffLinks version 2.1.1 [17], [18] without a reference transcriptome. CuffLinks was run using default parameters except for –no-effective-length-correction that was used to avoid overestimating expression of shorter isoforms of a gene. The CuffCompare module of CuffLinks was used to compare reconstructed transcripts to the ENSEMBL reference mouse GRCm38 transcriptome. FPKM (Fragments Per Kilobase of transcript per Million reads mapped) values for genes were generated using CuffDiff. FPKMs, gene names, genomic locations and gene types were extracted from genes.read_group_tracking, genes.fpkm_tracking and ENSEMBL GRCm38 reference file with a custom Python script into a single text file and further analyzed in MS Excel.

The trimmed raw sequencing data have been deposited in the NCBI Sequence Read Archive database under the accession code SRX499214.

### Primer Design

Reference mRNA sequences were obtained from the National Center for Biotechnology Information database (http://www.ncbi.nlm.nih.gov). To minimize the chance of amplification from contaminating gDNA, wherever possible we designed a primer pair with an intron located between forward and reverse primers. Specific oligonucleotide PCR primers were designed and selected using the Primer-Blast tool [19]. Each primer was compared to the entire GenBank nucleotide database to ensure that it recognizes only the gene of interest. For the quantitative PCR each pair of primers was validated to amplify only one product. The list of primers used in this study can be found in Table S1.

### PCR

Total RNA was converted to cDNA using High Capacity cDNA Reverse Transcription Kit (Applied Biosystems). For the detection, we used 100 ng of RNA and the final concentration of primers in each 20 µl PCR reaction was 150 nM. Non-reverse transcribed RNA was directly used in PCR reaction as a negative control when the risk of amplification from contaminating gDNA existed. The following cycling conditions were employed: 1 cycle at 50°C, 2 min; 1 cycle at 95°C., 5 min; 40 cycles at 95°C–0.5 min, 60°C–0.5 min, 72°C–0.5 min. For quantitative PCR all reactions were run in triplicates using Power SYBR Green PCR Master Mix and the ABI 7900HT Real-Time PCR System (Applied Biosystems). Results were normalized to the endogenous control Gapdh RNA.

To confirm the identity of Olfr558 we used total cornea cDNA, a pair of specific, intron-spanning primers – 5′-GGGGAAAAGACACACAGGCT-3′ (forward) and 5′-AGCCAGCCAAAACTGAACCT-3′ (reverse) and GoTaq Green Master Mix (Promega) to amplify a 169 bp DNA fragment. The resulting PCR product was isolated after agarose gel electrophoresis, blunt-ended by T4 DNA polymerase, phosphorylated by polynucleotide kinase, and ligated into linearized pcDNA3 vector. Plasmid DNA containing an insert was sequenced. Successful amplification from genomic DNA was to result in a 6809 bp fragment and would be impossible using the cycling conditions.

### *In situ* RNA Hybridization

*In situ* RNA hybridization was performed using RNAscope technology (Advanced Cell Diagnostics, Hayward, California) following the manufacturer’s protocol with minor modifications. Briefly, formalin fixed paraffin embedded total mouse eyes were cut into 5 µm sections and mounted on SuperFrost Plus glass slides. After de-paraffinization the slides were subjected to RNAscope Multiplex Fluorescent Assay. The procedure began with 15 min boiling in Pretreat 2, followed by pretreatment 3 (protease) for 30 min at 37°C. To reduce a potential background from hybridization of probes with chromosomal DNA we introduced an additional step – a treatment with DNase. After pretreatment 3 the slides were washes 5x with water, and a solution of DNase I (50 u/ml in 1x DNase I buffer, Ambion) was added to the eye tissue. To demonstrate that the signal comes from hybridization of probes with mRNA, some slides were treated with a mixture of DNase I and RNase A (5 mg/ml). The treatment was for 40 min at 37°C. At the end of the DNase treatment the slides were washed 5x with water, hybridized with RNAscope probes for 2 h at 40°C and the remainder of the assay protocol was implemented. The fluorescent signal was visualized and captured using an open-field Nikon Eclipse TE2000-U microscope. According Advanced Cell Diagnostics, each mRNA molecule hybridized to a probe appears as a separate small fluorescent dot.

### Immunohistochemistry

Immunostaining of eye sections with an antibody against α-smooth muscle actin (SMA) was performed following RNA *in situ* hybridization with the Olfr558 probe. *In situ* hybridization was done as described above with one modification: pretreatment 3 (protease) was for 20 min at room temperature. Following the last slide washes at the end of the RNAscope assay protocol, the eye sections were incubated with a blocking solution (10% BSA, 400 u/ml rRNasin in PBS) for 1 h at room temperature. The sections were then incubated with the anti-SMA antibody (rabbit polyclonal anti-mouse, Abcam ab32575) at 1∶200 dilution in the blocking solution with 0.1% Tween 20 overnight at 4°C. The slides were washed 3x for 15 min with PBS/0.1% Tween 20 (PBST) and incubated with a FITC-labeled anti-rabbit secondary antibody (Amersham Biosciences) at 1∶200 dilution in PBST for 1 h at room temperature. The slides were then washed 3x for 15 min with PBST and mounted.

To our knowledge, this is the first reported example of combining RNAscope assay with immunostaining. It is worth noting that immunostaining following the RNAscope assay may be difficult to implement because the *in situ* hybridization step involves proteolytic treatment of the tissue slice, which might destroy the antigen of interest. We used the antibody against SMA, which is an abundant protein expressed in smooth muscle cells supporting the vessel walls, and there was enough remaining antigen to visualize this protein.

## Results

### Analysis of a Corneal Transcriptome

Sequencing of total mouse cornea RNA resulted in over 46 million reads in each direction. The alignment of these reads to the mouse genome yielded 13,697 annotated transcripts with FPKM values of 5 or above. This number is similar to the number of transcripts reported for other tissues, such as the mouse trigeminal ganglia (12,984 gene products) and dorsal root ganglia (13,195) [20], and the human liver (17,396) [21]. To validate quality of the generated transcriptome we looked at genes that should or should not be expressed in the cornea. Keratin 12 (*Krt12*), which is known to be highly and selectively expressed in the corneal epithelium, and opticin (*Optc*), an extracellular matrix-associated protein specifically expressed in the eye, had FPKM values 2125 and 28, respectively. Conversely, insulin transcripts (*Ins1* and *Ins2*) and rhodopsin (*Rho*) were not found in the transcriptome. Thus, we found the genes expected to be in the cornea and did not find genes expected to be absent, confirming good quality and specificity of the generated corneal transcriptome profile.

### GPCRs in the Cornea

We then determined which GPCR transcripts were present in the corneal transcriptome. In total, transcripts for 233 non-olfactory GPCR genes were identified, which is similar to the number of GPCR genes reported expressed in the mouse trigeminal (202) or dorsal root ganglia (204) [20]. Of these, 98 had relatively high expression (FPKM ≥20, Table S2), representing almost a quarter of the 446 non-olfactory, non-pheromone receptor genes predicted to be present in the mouse genome [12]. Except for the class A subfamilies A16 (opsins) and A3 (angiotensin II and bradykinin receptors), all GPCR classes and their subfamilies were represented in the mouse corneal transcriptome. Among the most abundant were transcripts for several receptors with unknown functions – Gpr174 (FPKM 511), Gpr157 (FPKM 186), Gpr173 (FPKM 168). The cornea is one of the most innervated areas of the body with numerous nerve endings. Not surprisingly, we found several GPCRs that are normally present in neurons and involved in neuronal signal transduction, such as metabotropic glutamate receptor 8 (Grm8, FPKM 182), cannabinoid receptor type 1 (Cnr1, FPKM 191) and substance P receptor (Tacr1, FPKM 107). Several receptors that are believed to be involved in cell adhesion also appear to be highly expressed in the cornea, including latrophilin-3 (Lphn3, FPKM 187) and Gpr144 (FPKM 423). We also identified several GPCRs involved in the immune response, of which the most abundant was C-C chemokine receptor type 4 (CCR4, FPKM 260) – a receptor known to play a role in the allergic reaction in the eye and other organs.

### Olfactory Receptors in the Cornea

Surprisingly, in addition to other GPCRs, we found many genes assigned to the Olfr family. The corneal transcriptome contained sequences for 96 Olfrs with FPKM values 20 or above (Table S3). We did not find, however, any transcripts from two other large mouse GPCR families – V1R and V2R (vomeronasal receptors). These results indicate that while V1R and V2R families have no function in the cornea, Olfr expression is likely to be relevant.

### Validation of NGS Results by RT-PCR

**Figure 1:**
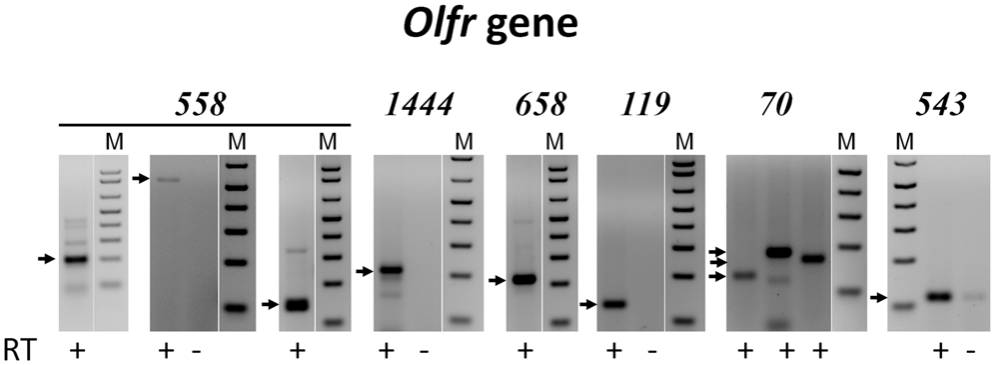
RT-PCR analysis of Olfr gene expression in the cornea. RT-PCR was performed as described in Materials and Methods using mouse corneal RNA and primers for the indicated Olfr genes. For primer pairs located within a single exon PCR was performed with (+) and without (–) the RT step. For primer pairs located in separate exons only results of PCR with (+) RT are shown. Arrows indicate the positions of the predicted PCR fragment sizes for *Olfr558* (200, 529 and 169 bp), *Olfr1444* (224 bp), *Olfr658* (220 bp), *Olfr119* (131 bp), *Olfr70* (133, 189 and 171 bp) and *Olfr543* (130 bp). Molecular markers (M) are from a 100 bp DNA ladder with the lowest band of 100 bp.

**Figure 2:**
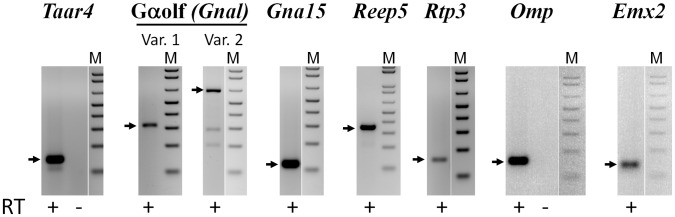
Expression of G protein subunits and other olfactory genes in the cornea. The RT-PCR analysis of corneal RNA was performed as described in Fig. 1 legend. For primers located within a single exon PCR was performed with (+) and without (–) the RT step. For primer pairs located in separate exons only results of PCR with (+) the RT are shown. Arrows indicate the positions of the predicted PCR fragment sizes for *Taar4* (140 bp), two splice variants of Gαolf (314 and 607 bp), *Gna15* (118 bp), *Reep5* (347 bp), *Rtp3* (180 bp), *Omp* (150 bp) and *Emx2* (133 bp). Molecular markers (M) are from a 100 bp DNA ladder; the lowest band is 100 bp.

To confirm the identified Olfr gene expression in the cornea we isolated RNA from independent samples and performed RT-PCR. Due to the large number of transcripts identified by NGS we analyzed a representative group of 18 genes. One of the potential problems in identifying GPCR transcripts by RT-PCR is that the majority of them are encoded by a single exon. Even a small contamination of the sample with gDNA could yield PCR bands that appear as transcript products. For this reason, we focused on Olfrs encoded by two or more exons, which allows distinguishing products derived from gDNA and mRNA by designing primers with an intron in-between. We also analyzed some single exon genes, performing the reaction with and without the reverse transcription step. Figure 1 shows results of RT-PCR for several confirmed Olfrs in the cornea, and Table 1 summarizes all data. For example, the presence of Olfr558 transcripts was tested with three different primer pairs (two of which are separated by an intron), with all three reactions generating products of the expected size. We also confirmed the presence of transcripts of two members of the second olfactory receptor family, trace amine associated receptors (TAAR) – Taar4 (Fig. 2) and Taar9. As a negative control, we tested Olfr312 and 643 that were not found in the corneal transcriptome (FPKM<5), and neither produced a PCR band (data not shown). However, about 50% of transcriptomics “hits” were not confirmed by RT-PCR: *e.g.*, out of 16 tested Olfrs detected in the transcriptome, 8 were confirmed by RT-PCR (Table 1).

**Table 1:** Olfactory receptor gene transcripts detected in the cornea by RT-PCR.

Gene name	Alt name	FPKM	predicted exons	Foundexons	RT-PCR	humanhomolog	% identity
Olfr119	MOR263-7	41	2	1	yes	none	
Olfr1444	MOR202-4	39	1	1	yes	OR5B21	86%
Olfr424	MOR105-2	26	1	1	yes	OR6K3	78%
Olfr543	MOR42-2	28	1	1	yes	none	
Olfr558	MOR18-1	22	2	1	yes	OR51E1	94%
Olfr658	MOR34-5	23	2	1	yes	OR52N4	89%
Olfr521	MOR101-2	27	1	1	yes	none	
Olfr633	MOR12-5	37	1	1	yes	none	
Olfr70	MOR262-10	24	2	1	yes	none	
Olfr167	MOR272-1	27	1	1	no	OR2L2	80%
Olfr332	MOR284-2	27	2	1	no	none	
Olfr370	MOR267-16	30	1	1	no	OR10K2	81%
Olfr524	MOR103-14P	34	1	1	no	none	
Olfr630	MOR17-1	36	2	1	no	none	
Olfr644	MOR13-1	29	1	1	no	none	
Olfr646	MOR33-2	54	1	1	no	OR52D1	89%
Olfr67	MOR31-1	20	2	1	no	none	
Olfr180	MOR184-9	22	2	1	no	OR5K4	78%
Olfr312	MOR222-4P	4	1	1	no	none	
Olfr643	MOR13-2	0	1	0	no	none	

Predicted exons –number of gene exons predicted by bioinformatics methods.

Found exons –number of exons from which transcripts were found after NGS.

RT-PCR – the expression of the gene was (yes) or was not (no) confirmed by RT-PCR; see Materials and Methods for details.

% identity – the percentage of identical amino acids in the mouse protein and its human homolog.

### Other Olfactory Signaling Genes in the Cornea

In addition to GPCRs we identified transcripts for several other genes involved in GPCR and olfactory signaling (Table 2) and confirmed their expression by RT-PCR (Fig. 2). Most notably, we found transcripts for both splice variants of the G protein associated with olfaction – Gαolf (*Gnal* gene). Transcripts for RTP3 and Reep5, members of protein chaperone families involved in Olfr folding and trafficking [22], were also identified. Emx2, a transcription factor shown to control transcription of a quarter of Olfr genes [23], is also expressed in the cornea. Interestingly, we found transcripts for olfactory marker protein, OMP. Whereas the function of OMP remains elusive, it is known to be highly and selectively expressed in the olfactory epithelium and olfactory bulb of the brain [24], [25]. OMP was shown to enhance olfactory signaling, possibly through aiding in maturation of olfactory neurons [26].

**Table 2:** GPCR/Olfr related genes found in the corneal transcriptome.

Gene name	Description	FPKM	RT-PCR
Taar4	Trace amine associated receptor	35	yes
Taar9	Trace amine associated receptor	23	yes
Gnas	G protein α subunit	21	-
Gnal	G protein α subunit - olfactory	54	yes
Gnai2	G protein α subunit	26	yes
Gna15	G protein α subunit	65	yes
Gnb1	G protein β subunit	43	-
Gnb5	G protein β subunit	0	no
Gng7	G protein γ subunit	31	-
RGS4	Regulator of G protein Signaling	41	yes
RGS12	Regulator of G protein Signaling	26	-
Adrbk1	β2-adrenergic receptor kinase	24	yes
REEP5	Receptor expression-enhancing protein	21	yes
RTP3	Receptor Trafficking Protein	35	yes
Adcy3	adenylyl cyclase	39	-
Cnga3	cAMP-gated channel	31	-
Cngb1	cAMP-gated channel	55	-
Omp	Olfactory Marker Protein	78	yes
Emx2	transcription factor	25	yes

RT-PCR – the expression of the gene was (yes) or was not (no) confirmed by RT-PCR, or not tested (−); see Materials and Methods for details.

### Quantitative PCR

**Figure 3:**
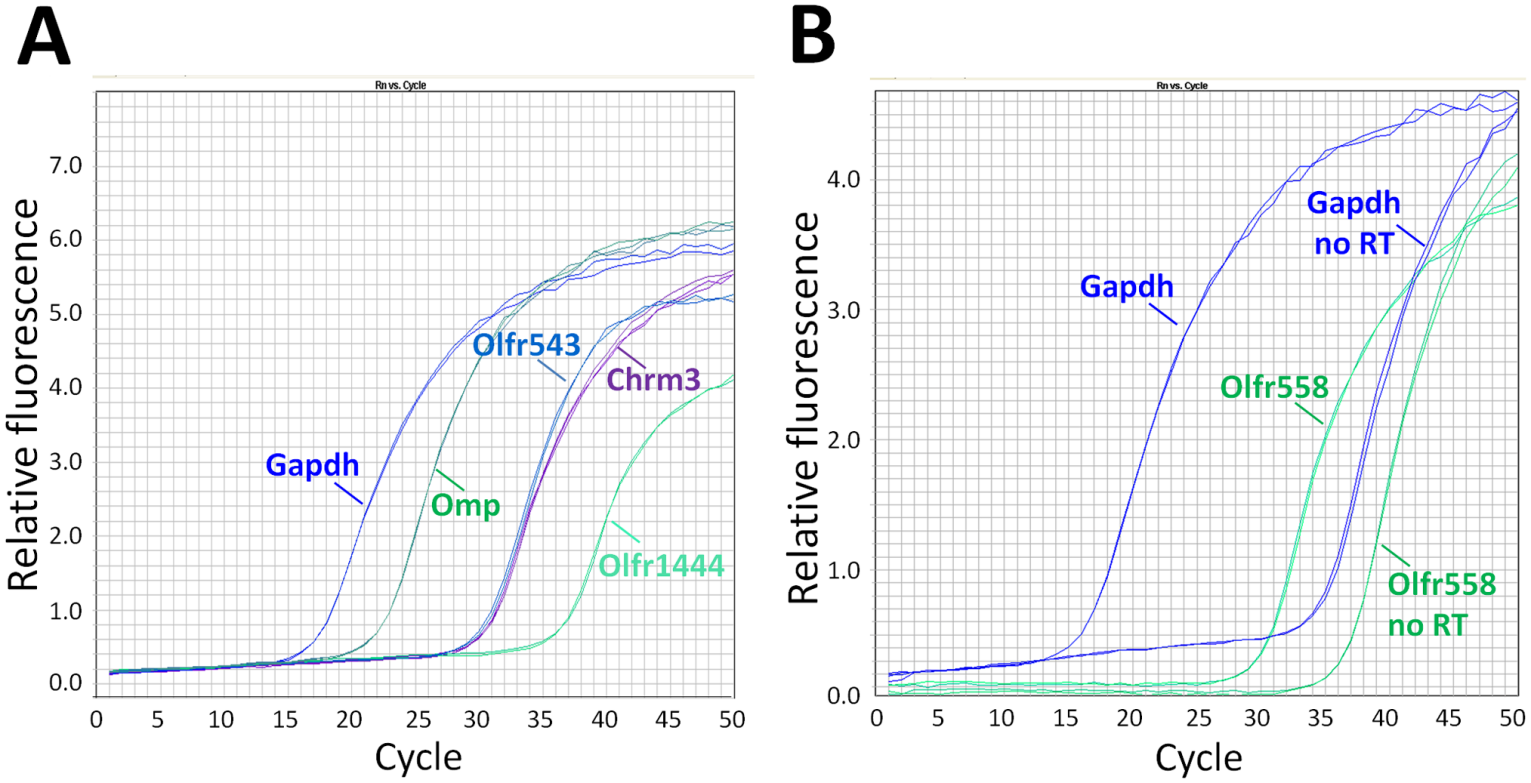
Quantitative PCR analysis of Olfrs and OMP. Corneal RNA was isolated and subjected to qPCR as described in Materials and Methods. PCR products for *Gapdh* and *Omp*, become detectable at earlier PCR cycles than products for genes expressed at lower levels, such as *Olfr543*, *Olfr1444* and *Olfr558*. Note that qPCR curves for *Olfr543* and *Chrm3* are virtually identical, suggesting that Olfr543 and M3-muscarinic receptor are expressed at similar levels in the cornea. When PCR primers were located within a single exon (*Gapdh* and *Olfr558*, Fig. 3B) PCR products were detected even in the absence of the RT reaction. However, they were detected at much later cycles compared to the reactions with the RT step, thus, contributing less than 2% to the total PCR product.

We performed qPCR using corneal RNA and primers for some of the identified genes. Although qPCR method is truly quantitative only when one uses the same primer set, our data provide a rough idea about the relative abundance of tested mRNAs. Figure 3 shows that some Olfrs, such as *Olfr1444*, are expressed at very low levels (Average C_t_ = 36), whereas others (*e.g.*, *Olfr543*, Average C_t_ = 30, and *Olfr558*, Average C_t_ = 29) are expressed at the levels comparable to other GPCRs in the cornea (e.g., M_3_ muscarinic receptor, *Chr3*). When PCR primers were located within a single exon (*Gapdh* and *Olfr558*, Fig. 3B) PCR products were detected even in the absence of the RT reaction. However, they were detected at much later cycles compared to the reactions with the RT step, thus, contributing less than 2% to the total PCR product. Note that the difference in curves ±RT was significantly bigger for Gapdh compared to Olfr558 reflecting a much higher level of Gapdh mRNA expression, whereas the background (–RT) was relatively similar. Note also that OMP mRNA (Average C_t_ = 22) appears to be relatively abundant in the cornea compared to Olfr mRNAs.

### *In situ* Hybridization with OMP and Gαolf mRNA

**Figure 4:**
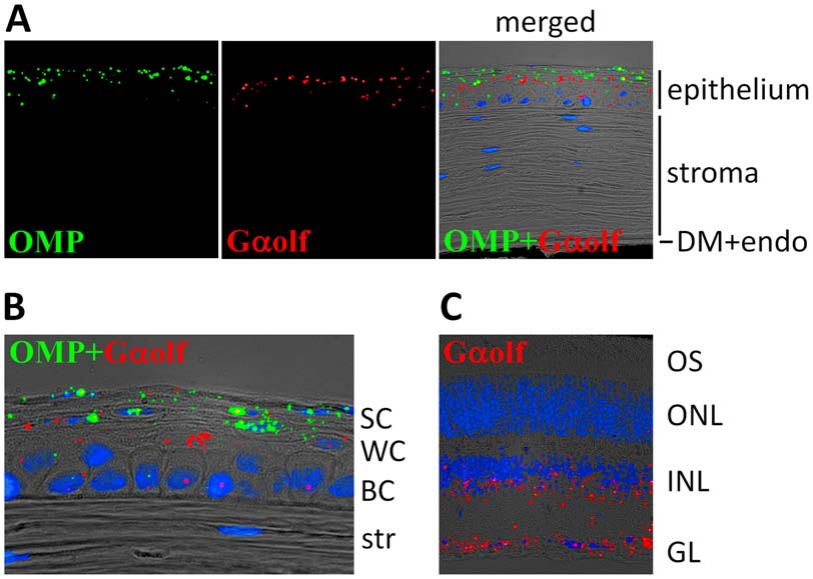
Localization of OMP and Gαolf expression in the mouse corneal epithelium. Imaging of corneal slices was performed as described in Materials and Methods. A. *In situ* hybridization with OMP (green) and Gαolf (red) probes. The merged image also shows bright field and DAPI staining (blue). The corneal layers are indicated on the right: epithelium, stroma with keratocytes and Descemet’s membrane with the endothelium (DM+endo). B. A higher magnification image of the corneal epithelium stained with OMP and Gαolf probes. Epithelial cell layers are indicated on the right – basal cells (BC), wing cells (WC) and superficial surface cells (SC); str – the stroma. Note that Gaolf is mostly expressed in wing cells, whereas OMP is mostly in superficial cells. C. Expression of Gαolf (red) in the mouse retina. Retinal cell layers are indicated on the right – ganglionic layer (GL), inner nuclear layer (INL) and outer nuclear layer (ONL); OS – photoreceptor outer segments.

To determine which cell types express OMP and Gαolf genes, we generated RNAscope probes for *in situ* mRNA hybridization. OMP signal was found only in the corneal epithelium (Fig. 4A), while no signal was present in the stroma, endothelium or other parts of the eye. Similarly, in the cornea, the mRNA for Gαolf was found only in the epithelium (Fig. 4A). There was an overlap of OMP and Gαolf signals, and some cells clearly co-express both genes. However, the distribution of the RNAscope signals marking these two gene products appear to be different across the corneal epithelium. Expression of Gαolf begins in the basal cell layer, peaks in the middle layer (wing cells) and goes down in the outermost superficial cell layer (Fig. 4B). The expression of OMP is seen only in very few cells in the basal cell layer, but becomes noticeable in the middle layer and peaks in the outermost superficial cell layer. In addition to the cornea, expression of Gαolf was also found in the retina, mainly in the ganglionic and inner nuclear layers (Fig. 4C). This pattern of expression is different from the nasal epithelium and olfactory bulb where OMP and Gαolf are co-expressed in the same olfactory neurons. It likely reflects the fact that OMP and Gαolf play distinct roles in signal transduction, which appear to be separated in the eye.

### Localization of Olfr558 mRNA

**Figure 5:**
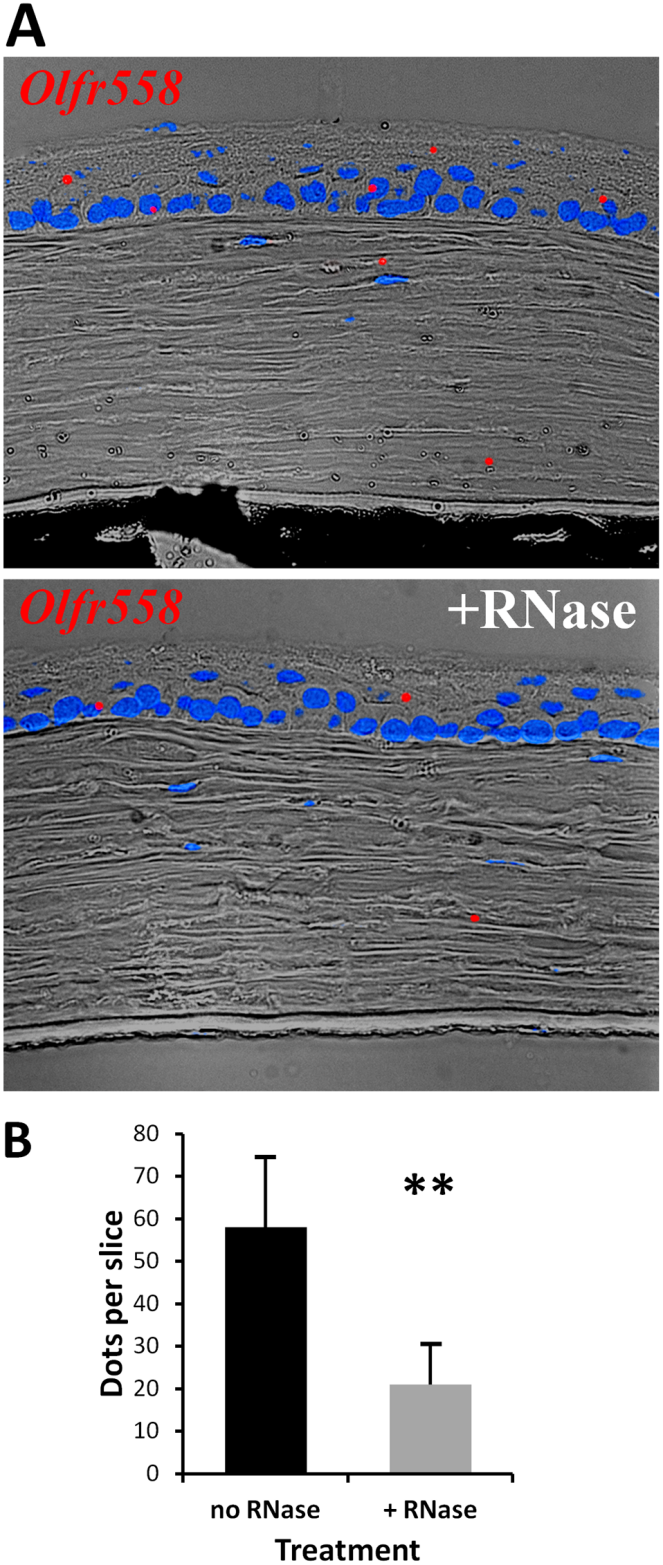
Expression of Olfr558 in the mouse cornea. A. Representative images of *in situ* hybridization of the mouse cornea slices with *Olfr558* probe (red) without or with the pretreatment with RNase (+RNase). DAPI (blue) was used for nuclei staining. B. Quantification of in situ hybridization signals obtained without or with RNase pretreatment. All signal dots were counted in the entire corneal slice for each condition, and the average number of dots per slice (n = 6) is shown. Asterisks indicate significant difference (p<0.01).

We selected Olfr558 to study its localization in the eye because earlier investigations identified butyric acid as a ligand for this receptor that activates it *in vitro* [13]. Whereas this may not necessarily be an endogenous agonist for this receptor, having such ligand allows performing follow-up functional studies. Furthermore, we confirmed the expression of *Olfr558* by RT-PCR with four primer pairs (Figures 1 and 3B) and sequencing of the PCR products. *In situ* hybridization with the mouse eye slices showed a relatively sparse labeling with RNAscope probe in the cornea (Fig. 5A). Pre-treatement with RNase significantly reduced the signal, indicated that much of this signal indeed comes from mRNA. On average, untreated slides contained significantly more dots per cornea than slides pre-treated with RNase (57±15 vs. 21±11, p<0.01; Fig. 5B).

**Figure 6:**
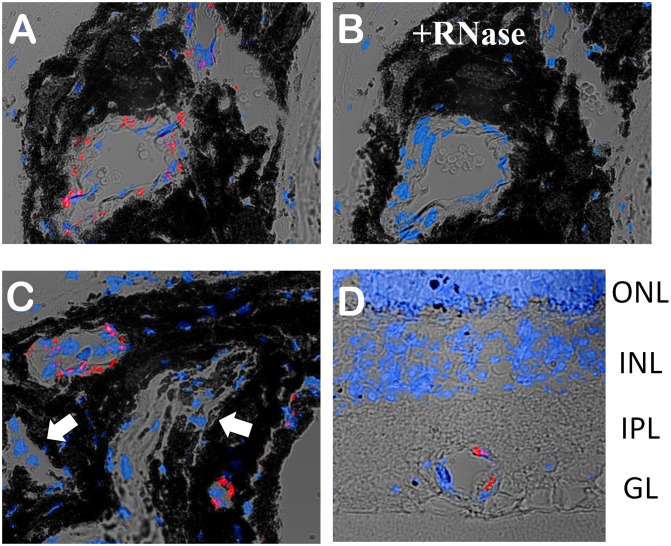
Expression of Olfr558 in vessels of the choroid and retina. A and B. *In situ* hybridization of the mouse choroid slices with *Olfr558* probe (red) without (A) or with (B) the pretreatment with RNase (+RNase). The slices in A and B were adjacent to each other in the eye. Blue – nuclei staining (DAPI). C. *In situ* hybridization of a different choroid slice with *Olfr558* probe (red). Note that not all vessels express *Olfr558*; some vessels do not have *Olfr558* signal at all (white arrows). D. *Olfr558* is also expressed in a few vessels of the mouse retina. The retinal layers are indicated on the right – ganglionic layer (GL), inner plexiform layer (IPL), inner nuclear layer (INL) and outer nuclear layer (ONL).

We noticed that fluorescent dots revealed by the Olfr558 probe where more abundant in choroid of the eye where they localized to blood vessels (Fig. 6A). Pre-treatment with RNase virtually eliminated the Olfr558 signal (Fig. 6B), indicating specificity of hybridization with RNA. Although less frequently than in the choroid, Olfr558 expression was also found in the vessels of the retina (Fig. 6D).

**Figure 7:**
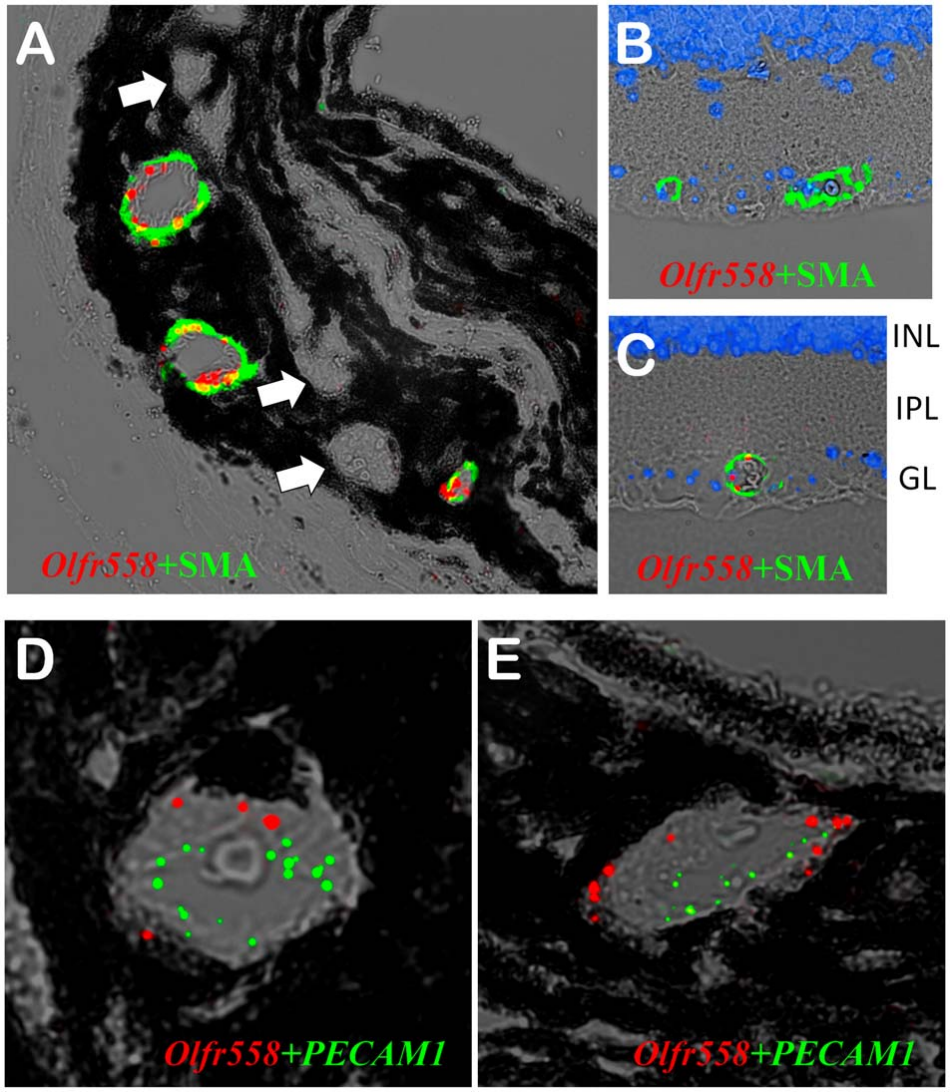
Co-localization of Olfr558 with α-smooth muscle actin in vessels. *In situ* RNA hybridization with *Olfr558* probe (red) was followed by immunostaining with an anti-SMA antibody (green), as described in Materials and Methods. A. Staining of the mouse choroid. All vessels that have *Olfr558* signal are also stained with the anti-SMA antibody, whereas the vessels without *Olfr558* signal are also devoid of SMA staining (white arrows). B and C. Staining of the mouse retina. Panel B shows a representative image where the SMA-positive vessels do not have *Olfr558* signal; the majority of retinal vessels to do not express *Olfr558* (see text). (C) A representative vessel from the same retinal slice that co-expresses *Olfr558* and SMA. The retinal layers are indicated on the right – ganglionic layer (GL), inner plexiform layer (IPL) and inner nuclear layer (INL). D and E. *In situ* hybridization of the mouse choroid with probes for *Olfr558* (red) and a marker of vessel endothelial cells *PECAM1* (CD31, green). Note that *PECAM1* puncta are located in the inner-most cell layer of the vessels, whereas *Olfr558* puncta are present in the outer layers.

Not all vessels in the choroid and retina showed Olfr558 expression (Fig. 6C). To identify the specific type of vessels expressing Olfr558, we performed double staining: *in situ* hybridization with the Olfr558 probe followed by immunostaining with an anti-α-smooth muscle actin (SMA) antibody. In the eye, SMA is mostly found in arteries and arterioles, whereas veins, venules and lymphatic vessels express much less of it or none at all [27]. As shown in Fig. 7A, the vessels positive for Olfr558 were also stained for SMA, showing almost complete co-localization in the choroid. The picture was somewhat different in the retina, where most vessels stained with for SMA had no Olfr558 signal (Fig. 7B), but the few Olfr558-positive vessels were also positive for SMA (Fig. 7C). We also performed double staining of the mouse choroid with RNAscope probes for Olfr558 and a marker of vessel endothelial cells, *PECAM1* (CD31 antigen). Whereas *PECAM1* puncta were located in the inner-most cell layer of the vessels, Olfr558 puncta were present in the outer layers, indicating that Olfr558 gene is expressed in vessel supportive smooth muscle cells and not in the endothelium (Fig. 7D and E).

## Discussion

### GPCRs in Corneal Transcriptome

This study for the first time provides experimental evidence for expression of multiple GPCRs and related signaling proteins in the cornea. Among the GPCR transcripts identified through NGS, we found nearly 100 transcripts encoding putative Olfrs. These genes were expressed at levels (FPKM values of ∼20–40) comparable to those for such classical GPCRs as muscarinic and adrenergic receptors (Table S1).

Since most GPCRs are encoded by mono-exonic genes, even miniscule contamination with genomic DNA could generate NGS reads that appear as their transcripts. Noteworthy, a given GPCR can be expressed in a small cell sub-population, whereas gDNA originates from all cells in the tissue. To improve the RNA isolation protocol, we added an additional DNase treatment step performed in solution. However, even after this step, some of our PCR reactions without RT amplified trace amounts of products of expected size (data not shown). Since the Illumina NGS protocol includes a PCR amplification step this underscores the necessity to confirm transcriptomics results by other methods. We selected 16 Olfrs from corneal transcriptome for confirmation with RT-PCR, and 8 of them were confirmed. In contrast, the confirmation rate for other (poly-exonic) genes was nearly 100% (Table 2). Thus, it appears that the relatively high rate of false positives for Olfrs found in the transcriptome is due to their low expression and the strong effect of genomic DNA contamination on mono-exonic genes.

The discovery of Olfr gene expression in the eye was surprising but not completely unexpected. In fact, fewer than 100 out of ∼1100 known mouse Olfrs were clearly demonstrated to play a role in sensing smell [13]. The remaining ∼1000 members were assigned to the Olfr family on the basis of sequence homology, and therefore should be referred to as putative Olfrs. While many of them are probably indeed involved in sensing odors, it is likely some of these receptors do not participate in olfaction and play different roles in physiology. In fact, a growing number of examples shows expression of putative Olfrs outside of the nasal cavity – in the heart, kidney, skeletal muscle, prostate and sperm [14]. While the function of Olfrs in these parts of the body is not well established, there are indications they may be involved in skeletal muscle regeneration after injury [28] and in sensing products of bacterial metabolism in the gut [29]. We speculate that Olfrs may play a similar function in the cornea. If pathogenic microbes produce metabolites that are different from the ones produced by commensals, Olfrs could recognize such molecules and aid in activating appropriate defense mechanisms. Such metabolites are likely to be small in size and able to diffuse from the surface of the eye into deeper layers of the cornea. Olfrs evolved to recognize small ligands and are perfectly suited for such function.

### Expression of Olfr558 in the Eye

We selected Olfr558 as the focus of our initial investigation for the following reasons. First, this gene consists of two exons, which makes RT-PCR results more reliable. Indeed, Olfr558 expression in the cornea was confirmed by RT-PCR using four different primer pairs (Figures 1 and 3B) and also by sequencing of the PCR product. Second, this is one of the few Olfrs that have a known agonist (butyric acid) activating it *in vitro* [13], which provides an opportunity for future functional studies. Third, Olfr558 also has a very close homolog in humans, OR51E1 (Table 1). It is likely Olfr558 and OR51E1 play similar functions in respective species. We therefore chose Olfr558 for RNA *in situ* hybridization analysis (Fig. 6, 7). We applied the recently developed RNAscope technology, which allows detection of a single RNA molecule with very high specificity [30].

Consistent with FPKM values and RT-PCR (Table 1, Fig. 3B), *in situ* hybridization showed that Olfr558 is expressed in the cornea at much lower levels than Gαolf and OMP (compare Figures 4A and 5A). Yet, RNAse treatment significantly reduced the number of fluorescent puncta per cornea revealed with the Olfr558 probe, indicating specificity of the signal. It is known that in the nasal epithelium, only a small fraction of all olfactory neurons express a particular receptor gene. Each olfactory neuron expresses only one kind of Olfr, and expression of other Olfrs is suppressed [31]. If this “one cell, one receptor” paradigm is universal, one should expect only a small fraction of corneal cells to express Olfr558. The downstream signaling components, e.g., Gαolf and other related genes are present in all olfactory neurons, and so their overall expression level is higher. At the moment we do not know what specific role Olfr558 plays in the cornea. It was reported that the addition of butyric acid to cultured rabbit corneal blocks *in situ* stimulated fibronectin synthesis [32]. Fibronectin plays an important role in corneal wound healing. Since butyric acid can activate Olfr558, we are tempted to speculate that Olfr558 could be involved in wound healing, similarly to what was reported about Olfr16 (MOR23) and skeletal muscle healing [28].

We detected a more pronounced signal for Olfr558 mRNA in the vessels of the choroid and retina. Not all vessels express Olfr558, underscoring specificity of labeling. About 50% of vessels in the choroid and 10–20% in retina showed the Olfr558 signal. To determine what type of vessels express Olfr558 we performed double staining with Olfr558 RNAscope probe and an antibody against α-smooth muscle actin. In the eye SMA is mainly expressed in arteries and arterioles, whereas veins, venules and lymphatic vessels express little or no SMA [27]. In the choroid, Olfr558 and SMA signals were virtually completely co-localized, whereas in the retina only a fraction of the SMA-positive vessels expressed Olfr558. Thus, our results suggest that Olfr558 is more important for blood flow in the choroid than in retina. Smooth muscle cells regulate the caliber of the blood vessels by contracting or relaxing in response to appropriate stimuli. One of the receptors causing vasodilation (β_2_-adrenergic) has a similar downstream cascade to that of Olfrs: the highly homologous G protein alpha, Gαs and adenylyl cyclase, which increases cAMP production. It is conceivable that activation of Olfr558 by its ligand could cause the same effect as activation of β2-adrenergic receptor (vasodilation), increasing the blood flow and delivery of oxygen and nutrients in the eye. Interestingly, a recent report showed that a different Olfr, Olfr78, is also expressed in vascular smooth muscle but in different parts of the body – the kidney, heart and skeletal muscle [29]. The authors demonstrated that propionic acid, an agonist for Olfr78, caused vasodilation and a drop in blood pressure in mice. Olfr78 is the closest relative of Olfr558 in mice and it would not be surprising if these receptors played a similar function in different parts of the body. The choroid vessels play an important role by providing oxygen and nourishment to the outer layers of the retina. We can speculate that Olfr558 is involved in regulation of the arteriole diameter and blood flow in the choroid under certain normal or pathophysiological conditions. The fact that OR51E1 is a very close homolog of Olfr558 in humans (94% identity) suggests these receptors share the same ligands and that OR51E1 serves the same function in humans as Olfr558 in mice. We did not find any signal for Gαolf in vessels of either the choroid or retina, suggesting that Olfr558 in vessels couples to a different G protein, most likely Gαs.

### Olfactory Pathway Gene Expression in the Eye

Although the function of Olfrs in the eye is unknown, expression of other genes involved in olfactory signaling (Table 2, Figs 2–4) strongly supports the idea that Olfr-mediated signaling occurs in ocular tissues. For example, transcription factor Emx2, which we found in the cornea, controls expression of at least a quarter of Olfr genes in the nasal epithelium [23]. Protein chaperones RTP3 and REEP5 aid in receptor folding and trafficking to the surface [22]. It is reasonable to expect that these gene products play a similar role in the eye.

Olfactory-specific G protein alpha subunit Gαolf transmits signals from Olfrs to downstream effectors [33]. It was thought to be expressed only in the olfactory epithelium and the olfactory bulb [34], but later Gαolf was shown to be also expressed in other parts of the brain [35]. Moreover, a recent report showed its expression in the kidney as well [36]. It is very likely that Gαolf expressed in the cornea couples activated Olfrs to the downstream signaling cascade involved in corneal physiology. Our data show that Gαolf is also expressed the ganglionic and inner nuclear layers of the retina (Fig. 4), suggesting that there could be other Olfrs expressed in the retina.

Another gene strongly associated with olfaction is OMP, which initially was found only in the olfactory epithelium and olfactory bulb [24], [25]. However, later studies demonstrated that OMP is also expressed, albeit at much lower levels, in a small number of cells in other parts of the brain [37]. The function of OMP is yet to be established, but OMP gene knockout mice have a reduced ability to smell [38]. A recent study suggests that OMP may not directly interact with Olfrs but might be involved in terminal differentiation and maturation of olfactory neurons after birth in mice [26]. In the cornea basal cells give rise to wing cells, which differentiate further, flatten and become superficial cells, where OMP is mostly expressed (Fig. 4). Since no OMP was found in other parts of the eye, we can speculate that OMP might be specifically involved in the process of corneal epithelial cell development.

In summary, this study utilized NGS to provide a framework for systematic analysis of GPCRs in the cornea. Our experiments provided strong evidence for the presence of a novel class of GPCRs in the eye, putative olfactory receptors. While the full list of genes found in transcriptome requires validation, we confirmed the expression of a subset of genes by alternative methods. The agonists and functions of ocular Olfrs also remain to be established, however, restricted expression pattern and well-known drugability of GPCR superfamily identify them as a novel therapeutic target in ocular diseases.

## Supporting Information

Table S1 **PCR primers.**
(XLSX)

Table S2 **GPCR transcripts.**
(DOC)

Table S3 **Olfr transcripts.**
(DOC)
