# Supplementary material for: Expression of Olfactory Signaling Genes in the Eye
Source: PLoS One. 2014 Apr 30;9(4):e96435. doi: 10.1371/journal.pone.0096435 (PMC4005753; doi:10.1371/journal.pone.0096435)
Supplement: Table S2 — GPCR transcripts. (DOC) [file pone.0096435.s002.doc]

**Supplementary table 2**. – Non-olfactory GPCR transcripts found in the corneal transcriptome.

| **GPCR Class** | **Subfamily** | **Gene short name** | **FPKM** |
| --- | --- | --- | --- |
| Class A (1) Rhodopsin | 1 - Chemokine | Ccr4 | 260 |
|  |  | Ccr1l1 | 63 |
|  |  | Ccr2 | 29 |
|  |  | Ccrl1 | 28 |
|  |  | Ccr9 | 27 |
|  | 2 - Chemokine | Cxcr6 | 39 |
|  |  | Cxcr7 | 34 |
|  |  | Cxcr2 | 30 |
|  | 4 - Opioid | Oprm1 | 71 |
|  |  | Oprk1 | 35 |
|  | 5 - Galanin | Rxfp2 | 86 |
|  |  | Galr2 | 42 |
|  |  | Rxfp3 | 37 |
|  |  | Cysltr1 | 31 |
|  | 6 - Hypocretin | Hcrtr2 | 29 |
|  | 7 - Endothelin | Trhr | 86 |
|  |  | Ntsr1 | 80 |
|  |  | Ednra | 51 |
|  |  | Gpr39 | 20 |
|  | 8 - Formyl peptide | C3ar1 | 49 |
|  |  | Fpr2 | 20 |
|  |  | Gpr77 | 26 |
|  | 9 - Tachykinin | Tacr1 | 107 |
|  |  | Npy6r | 56 |
|  |  | Prokr1 | 24 |
|  |  | Tacr2 | 20 |
|  |  | Gpr83 | 35 |
|  | 10 - Thyrotropin | Lgr4 | 103 |
|  |  | Lgr5 | 41 |
|  | 11 - P2 purinergic | P2ry6 | 85 |
|  | 12 - P2 purinergic | Gpr87 | 29 |
|  | 13 - Cannabinoid | Cnr1 | 191 |
|  |  | Lpar2 | 61 |
|  |  | S1pr3 | 32 |
|  |  | Cnr2 | 22 |
|  |  | Lpar1 | 22 |
|  | 14 - Prostaglandin | Ptger2 | 22 |
|  | 15 - Thrombin | Lpar6 | 66 |
|  |  | F2r | 49 |
|  | 17 - Adrenergic | Htr2c | 389 |
|  |  | Adra1a | 206 |
|  |  | Adrb3 | 65 |
|  |  | Htr3b | 64 |
|  |  | Htr2b | 51 |
|  |  | Taar4 | 35 |
|  |  | Adra1b | 35 |
|  |  | Taar9 | 23 |
|  | 18 - Muscarinic | Hrh1 | 35 |
|  |  | Chrm1 | 28 |
|  |  | Chrm3 | 20 |
|  |  | Gpr63 | 39 |
|  |  | Gpr21 | 32 |
|  |  | Gpr101 | 30 |
|  | 19 - Serotonin | Htr7 | 25 |
|  |  | Htr5a | 20 |
| Class B (2) Secretin |  | Calcr | 631 |
|  |  | Lphn3 | 187 |
|  |  | Lphn2 | 83 |
|  |  | Celsr2 | 61 |
|  |  | Bai2 | 52 |
|  |  | Bai3 | 50 |
|  |  | Vipr2 | 30 |
|  |  | Celsr3 | 25 |
|  |  | Celsr1 | 23 |
|  |  | Gpr114 | 48 |
|  |  | Gpr110 | 33 |
|  |  | Gpr112 | 22 |
|  |  | Gpr128 | 177 |
|  |  | Gpr126 | 95 |
|  |  | Gpr123 | 25 |
|  |  | Gpr144 | 423 |
|  |  | Gpr116 | 34 |
| Class C (3) Glutamate |  | Grm8 | 182 |
|  |  | Grm7 | 103 |
|  |  | Grm4 | 43 |
|  |  | Grm1 | 41 |
|  |  | Tas1r2 | 38 |
|  |  | Tas1r1 | 32 |
|  |  | Gprc5d | 22 |
| Class F (6) Frizzled |  | Smo | 31 |
|  |  | Fzd6 | 29 |
|  |  | Fzd2 | 26 |
|  |  | Fzd1 | 22 |
| Other |  | Gpr174 | 511 |
|  |  | Gpr157 | 186 |
|  |  | Gpr173 | 168 |
|  |  | Gpr165 | 118 |
|  |  | Gpr26 | 115 |
|  |  | Gpr98 | 95 |
|  |  | Gpr149 | 78 |
|  |  | Gpr150 | 52 |
|  |  | Gpr133 | 45 |
|  |  | Gpr107 | 44 |
|  |  | Gpr89 | 35 |
|  |  | Gpr160 | 34 |
|  |  | Gpr137 | 31 |
|  |  | Gpr124 | 21 |
|  |  | Gpr183 | 20 |
