# Supplementary material for: Expression of Olfactory Signaling Genes in the Eye
Source: PLoS One. 2014 Apr 30;9(4):e96435. doi: 10.1371/journal.pone.0096435 (PMC4005753; doi:10.1371/journal.pone.0096435)
Supplement: Table S3 — Olfr transcripts. (DOC) [file pone.0096435.s003.doc]

**Supplementary Table 3 –** Olfactory receptor transcripts found in the corneal transcriptome.

| **Gene short name** | **FPKM** |
| --- | --- |
| Olfr613 | 192 |
| Olfr1420 | 180 |
| Olfr1258 | 165 |
| Olfr1346 | 142 |
| Olfr1098 | 124 |
| Olfr1271 | 118 |
| Olfr287 | 113 |
| Olfr206 | 98 |
| Olfr102 | 95 |
| Olfr231 | 93 |
| Olfr129 | 88 |
| Olfr735 | 81 |
| Olfr118 | 78 |
| Olfr679 | 76 |
| Olfr1416 | 76 |
| Olfr197 | 76 |
| Olfr94 | 61 |
| Olfr656 | 59 |
| Olfr1364 | 59 |
| Olfr59 | 57 |
| Olfr1055 | 56 |
| Olfr1418 | 56 |
| Olfr646 | 54 |
| Olfr18 | 54 |
| Olfr745 | 53 |
| Olfr1186 | 53 |
| Olfr1330 | 53 |
| Olfr716 | 52 |
| Olfr543 | 48 |
| Olfr395 | 45 |
| Olfr266 | 45 |
| Olfr734 | 44 |
| Olfr133 | 44 |
| Olfr482 | 44 |
| Olfr555 | 42 |
| Olfr119 | 41 |
| Olfr822 | 41 |
| Olfr294 | 40 |
| Olfr78 | 40 |
| Olfr1289 | 39 |
| Olfr140 | 37 |
| Olfr633 | 37 |
| Olfr740 | 37 |
| Olfr630 | 36 |
| Olfr653 | 36 |
| Olfr524 | 34 |
| Olfr678 | 34 |
| Olfr31 | 32 |
| Olfr61 | 32 |
| Olfr1290 | 31 |
| Olfr983 | 30 |
| Olfr370 | 30 |
| Olfr599 | 29 |
| Olfr644 | 29 |
| Olfr694 | 28 |
| Olfr62 | 28 |
| Olfr554 | 28 |
| Olfr135 | 28 |
| Olfr1251 | 28 |
| Olfr1317 | 27 |
| Olfr1443 | 27 |
| Olfr126 | 27 |
| Olfr521 | 27 |
| Olfr332 | 27 |
| Olfr998 | 27 |
| Olfr1013 | 27 |
| Olfr167 | 27 |
| Olfr360 | 26 |
| Olfr424 | 26 |
| Olfr1286 | 25 |
| Olfr523 | 25 |
| Olfr70 | 24 |
| Olfr654 | 24 |
| Olfr1392 | 24 |
| Olfr859 | 24 |
| Olfr1138 | 23 |
| Olfr658 | 23 |
| Olfr108 | 23 |
| Olfr888 | 23 |
| Olfr180 | 22 |
| Olfr536 | 22 |
| Olfr112 | 22 |
| Olfr558 | 22 |
| Olfr1246 | 22 |
| Olfr449 | 22 |
| Olfr1009 | 22 |
| Olfr1324 | 22 |
| Olfr1132 | 22 |
| Olfr701 | 22 |
| Olfr711 | 21 |
| Olfr986 | 21 |
| Olfr1162 | 21 |
| Olfr672 | 21 |
| Olfr569 | 21 |
| Olfr1444 | 20 |
| Olfr67 | 20 |
